# Hepatitis B infection and preeclampsia among pregnant Sudanese women

**DOI:** 10.1186/s12985-018-0927-5

**Published:** 2018-01-22

**Authors:** Mohamed A. Ahmed, Manal E. Sharif, Duria A. Rayis, Abubakr M. Nasr, Ishag Adam

**Affiliations:** 1 Faculty of Medicine, University of Khartoum, P. O. Box 102, Khartoum, Sudan; 2 Faculty of Medicine Al-Neelain University, Khartoum, Sudan

## Abstract

**Background:**

Previous published studies have reported conflicting results of association between hepatitis B virus (HBV) infection and preeclampsia. There was no published data on HBV and preeclampsia in Africa including Sudan. The aim of the present study was to investigate the association between HBsAg seropositivity and preeclampsia.

**Methods:**

A case –controls study (200 women in each arm) was conducted at Saad Abualila Maternity Hospital, Khartoum, Sudan.

The cases were women with preeclampsia and the controls were healthy pregnant women. Socio-demographic characteristics were gathered using questionnaire and HBsAg was investigated using an ELISA.

**Results:**

There was no significant difference between the cases and the controls in their age, parity, residence, education and blood groups. The majority of the cases were mild preeclampsia (159; 79.5%).

In comparison with the controls, a significantly higher number of the cases were HBsAg seropositive [30 (15.0%) vs.12 (6.0%), *P* = 0.005]. In binary regression women with HBsAg seropositive were at higher risk of preeclampsia than women who were HBsAg seronegative (OR = 2.86, 95%, CI = 1.41–5.79, *P* = 0.003).

**Conclusion:**

In the current study HBsAg seropositivity is associated with preeclampsia. Preventive measure should be implemented.

## Background

Preeclampsia is the occurrence of both hypertension (systolic blood pressure ≥ 140 mm HG or diastolic ≥ 90 mm HG) and proteinuria in the second half of pregnancy [1]. It has been estimated that there are around 8.5 million cases of preeclampsia reported worldwide annually and preeclampsia complicates around 2–4% of deliveries [2, 3]. Preeclampsia/eclampsia is a major health problem worldwide and it is the main cause of maternal and perinatal morbidity and mortality [2, 4]. Although the exact pathophysiology of preeclampsia is not yet fully understood, it is characterized by widespread maternal endothelial dysfunction which might be triggered by maternal infections. There is a growing body of evidence of association between various bacterial/viral maternal infections and preeclampsia [5, 6].

Previous published studies have reported conflicting results of association between maternal hepatitis B virus (HBV) infection and pregnancy induced hypertension. While some studies reported a significantly positive association between maternal HBV infection and pregnancy induced hypertension [7], some other authors observed no association or even negative association between HBV and preeclampsia [8–11].

Furthermore most of these studies have been done in USA [12], Europe and South East Asia [10, 11]. To our best knowledge, there was no published data on HBV and preeclampsia in Africa including Sudan. Moreover, the rate /incidence of preeclampsia and HBV varied between different ethnicities and different settings [13, 14]. Unlike the developed countries, the vast majority of African countries characterized by a high HBV disease endemicity [15]. Therefore, the observation of the previous studies might not be generalizable to our setting.

Investigating the association between HBV and preeclampsia is of paramount importance for clinicians and health planners because preventive measures and treatment could be employed to prevent preeclampsia and its undesirable adverse effects.

Preeclampsia/eclampsia is the major health problem and associated with high maternal and perinatal mortality in Sudan [16, 17]. Moreover we have previously reported that (5.6%) of pregnant Sudanese women were positive for HBsAg [18]. The current study was conducted to investigate the association between HBV and preeclampsia in Khartoum, Sudan.

## Methods

A case –controls study (200 women in each arm) was conducted at Saad Abualila Maternity Hospital, Khartoum, Sudan during the period of February through November 2016.

The cases were women with preeclampsia (blood pressure ≥ 140/90 mmHg on 2 occasions, at least 6 h apart, and proteinuria of ≥ 300 mg/24 h). The cases were classified as mild and severe preeclampsia which is defined by the occurrence of; blood pressure ≥ 160/110 mmHg on 2 occasions, at least 6 h apart and/ proteinuria of ≥ 5 g/24 h), HELLP syndrome “hypertension, proteinuria and presence of hemolytic anemia, elevated liver enzymes and low platelet count” [1].

The controls were pregnant women without hypertensive disorders, nephropathy, or diabetes or any underlying disease. Both the cases and the controls had singleton pregnancy.

After signing an informed consent, medical and obstetrics history (age, parity, and gestational age) were gathered using a questionnaire. Five mL of blood was withdrawn, allowed to clot, centrifuged and tested for HBsAg using an ELISA (4th generation).

A sample size of 200 women in each arm of the study was calculated guided by the previous rate (5.6%) of pregnant Sudanese women were positive for HBsAg [18]. This sample size have over 80% power to detect a difference of 5% at α = 0.05. We assumed that 10% of the women might not respond or have incomplete data.

### Statistics

The collected data were analysed using SPSS V.20.0 (SPSS Inc., Chicago, IL, USA). Student’s t test and χ^2^ tests were used to compare continuous and categorized data respectively between the cases and the controls. Binary regression analyses were performed where preeclampsia was the dependent variable and expected risk factors (age, parity, education, etc.) and HBsAg seropositivity were the independent variables. Odds ratio (OR) with a 95% confidence interval (CI) was calculated and statistical significance was defined as *P* < 0.05.

## Results

There was no significant difference between the cases and the controls (200 women in each arm) in their age, parity, residence, education and blood groups, Table 1.

**Table 1:** Comparing socio-demographic characteristics between women with preeclampsia and controls in Saad Abualila Hospital, Khartoum, Sudan

Variable	Women with preeclampsia (*N* = 200)	Controls women (N = 200)	*P*
Mean (SD) of			
Age, years	27.9 (5.2)	27.1 (5.5)	0.128
Parity	2.1 (1.8)	1.8 (1.7)	0.76
Frequency (%) of			
Rural residence	140 (70.0)	144 (72.0)	0.741
Education ≤ secondary level	147 (73.5)	150 (75.0)	0.819
O blood group	103 (51.5)	105 (52.5)	0.422

The majority of the cases had mild preeclampsia (159; 79.5%).

In comparison with the controls, significantly a higher number of the cases were HBsAg seropositive [30(15.0%) vs.12 (6.0%), *P* = 0.005]. HBsAg seropositivity was different between the mild and severe cases [22/159(13.8%) vs. 8/41(19.5%), *P* = 0.461. None of the cases or the controls had jaundice or treated with antiviral drugs, e.g., nucleoti/side analogues.

In binary regression women with HBsAg seropositive were at higher risk of preeclampsia than women who were HBsAg seronegative (OR = 2.86, 95%, CI = 1.41–5.79, *P* = 0.003), Table 2.

**Table 2:** Binary regression analyses of the factors associated with preeclampsia in Saad Abualila Hospital, Khartoum, Sudan

Variable	Odd s ratio	95% confidence interval	*P*
Age	0.98	0.94–1.03	0.669
Parity	0.90	0.78–1.04	0.76
Rural residence	1.11	0.70–1.75	0.643
Education ≤ secondary level	1.04	0.62–1.75	0.857
O blood group	0.99	0.66–1.48	0.985
HBVs Ag positive	2.86	1.41–5.79	0.003

## Discussion

The main finding of the current study was the increased risk (2.86 times) of preeclampsia in HBsAg seropositive women. This is comparable to previous findings where Gargari et al., reported increased risk of pregnancy induced hypertension with maternal HBsAg seropositivity in a case-control study among pregnant Iranian women (450 HBV carriers and equal number of the controls, OR  =  4.2, 95%CI: 2.2, 8.1) [7].

In contrast two previous large studies were conducted in USA. One of these studies enrolled 297,664 subjects among whom 814 HBV carriers across 37 states using the discharge registry data from 1054 hospitals. Results demonstrated no association between maternal HBV infection and preeclampsia [12]. The other one enrolled 1, 670, 369 of whom 1458 HBV were carriers and again no significant association was observed between HBV and preeclampsia [19]. Likewise no significant association between maternal HBV infection and the risk of preeclampsia was found in many previous reports from various settings [8, 11, 12, 20]. The non-significant association (OR = 1.03, 95% CI: 0.78–1.35) between HBV infection and preeclampsia was recently reported in a metanalyses of fourteen studies [9].

Comparatively Lao et al. studied 8634 HBV carriers among 86,537 pregnant women in Hong Kong [18], Their results showed that maternal HBV infection was associated with reduced risk of both pregnancy induced hypertension (OR  =  0.79, 95%CI: 0.66, 0.95) as well as preeclampsia (OR  =  0.71, 95%CI: 0.56, 0.91) [11]. Interestingly a negative association between HBsAg carrier status and gestational hypertension was reported in the same region in a large sample study (13,792 women of whom 1340 (9.71%) were chronic HBsAg carriers) [10]. Furthermore a negative association (odds ratio = 0.77, 95% confidence interval, 0.65–0.90, *P* = 0.002) between chronic HBV infection and preeclampsia has been recently documented in a met -analysis which included 11,566 preeclamptic women in South East Asia [21]. Caution should be made when comparing our results with the later studies because of the difference in the study types and the different ethnic groups as well as difference in the immunological reaction to variable infections. Despite meticulous exclusion of confounders in most of these studies, the possibility of the bias still holds. Perhaps the care given to the patients vary from one centre to another or from one disease spectrum to another e.g. hypertensive patients with HBsAg positive could have been managed in a different settings that were primarily aimed for care of health pregnant women. It seems that the epidemiology and pathophysiology of preeclampsia is different in different regions. Parasitic [22], bacterial [5] and viral infections [23] were observed to be associated with preeclampsia in Sudan.

There are plausible explanations for the association between hepatitis and preeclampsia where previous studies have reported that HBV infection could increase the risk of atherosclerosis [24]. Secondly, preeclampsia (which is characterized by widespread maternal endothelial dysfunction) may be triggered by/result of an imbalance between angiogenic, anti-angiogenic and pro-angiogenic factors e.g. vascular endothelial growth factor [25, 26]. A significant association/ correlation between HBV, insulin resistance, thrombocytopenia, obesity and renal injury/proteinuria has recently been reported [27–29]. The interaction between these diseases and HBV might explain the association between HBV and preeclampsia.

One of the limitations of the current study was that some factors e.g. smoking and alcohol consummation were not investigated. Actually (according to the tradition) we were concerned of participants’ co-operation if they were asked about smoking and alcohol consummation and hence invalid results. However, a recent study reported the association between smoking, HBV and preeclampsia [8]. HBV genotype was not determined. It is expected that not only HBV E but also A and D is predominant in Sudan [30]. The enrolled women have different ethnic and genetic background/group which might have effects on both preeclampsia and HBV. Furthermore both HCV and I HIV were not investigated in this cohort of women. We have previously reported low rates of both HCV (0.6%) and HIV(1.0%) among pregnant Sudanese women [18, 31].

## Conclusion

In the current study HBsAg seropositivity is associated with preeclampsia. Preventive measure should be implemented. More research is need.
